# CAPTVRED: an automated pipeline for viral tracking and discovery from capture-based metagenomics samples

**DOI:** 10.1093/bioadv/vbae150

**Published:** 2024-10-08

**Authors:** Maria Tarradas-Alemany, Sandra Martínez-Puchol, Cristina Mejías-Molina, Marta Itarte, Marta Rusiñol, Sílvia Bofill-Mas, Josep F Abril

**Affiliations:** Computational Genomics Lab, Department of Genetics, Microbiology and Statistics, Universitat de Barcelona (UB), Institut de Biomedicina UB (IBUB), Barcelona, Catalonia 08028, Spain; Laboratory of Viruses Contaminants of Water and Food, Department of Genetics, Microbiology and Statistics, Universitat de Barcelona (UB), Barcelona, Catalonia 08028, Spain; Laboratory of Viruses Contaminants of Water and Food, Department of Genetics, Microbiology and Statistics, Universitat de Barcelona (UB), Barcelona, Catalonia 08028, Spain; Vicerectorat de Recerca, Universitat de Barcelona (UB), Barcelona, Catalonia 08007, Spain; Laboratory of Viruses Contaminants of Water and Food, Department of Genetics, Microbiology and Statistics, Universitat de Barcelona (UB), Barcelona, Catalonia 08028, Spain; The Water Research Institute (IdRA), Universitat de Barcelona (UB), Barcelona, Catalonia 08007, Spain; Laboratory of Viruses Contaminants of Water and Food, Department of Genetics, Microbiology and Statistics, Universitat de Barcelona (UB), Barcelona, Catalonia 08028, Spain; The Water Research Institute (IdRA), Universitat de Barcelona (UB), Barcelona, Catalonia 08007, Spain; Laboratory of Viruses Contaminants of Water and Food, Department of Genetics, Microbiology and Statistics, Universitat de Barcelona (UB), Barcelona, Catalonia 08028, Spain; The Water Research Institute (IdRA), Universitat de Barcelona (UB), Barcelona, Catalonia 08007, Spain; Laboratory of Viruses Contaminants of Water and Food, Department of Genetics, Microbiology and Statistics, Universitat de Barcelona (UB), Barcelona, Catalonia 08028, Spain; The Water Research Institute (IdRA), Universitat de Barcelona (UB), Barcelona, Catalonia 08007, Spain; Computational Genomics Lab, Department of Genetics, Microbiology and Statistics, Universitat de Barcelona (UB), Institut de Biomedicina UB (IBUB), Barcelona, Catalonia 08028, Spain

## Abstract

**Summary:**

Target Enrichment Sequencing or Capture-based metagenomics has emerged as an approach of interest for viral metagenomics in complex samples. However, these datasets are usually analyzed with standard downstream Bioinformatics analyses. CAPTVRED (*Capture-based metagenomics Analysis Pipeline for tracking ViRal species from Environmental Datasets*), has been designed to assess the virome present in complex samples, specially focused on those obtained by Target Enrichment Sequencing approach. This work aims to provide a user-friendly tool that complements this sequencing approach for the total or partial virome description, especially from environmental matrices. It includes a setup module which allows preparation and adjustment of the pipeline to any capture panel directed to a set of species of interest. The tool also aims to reduce time and computational cost, as well as to provide comprehensive, reproducible, and accessible results while being easy to costume, set up, and install.

**Availability and implementation:**

Source code and test datasets are freely available at github repository: https://github.com/CompGenLabUB/CAPTVRED.git

## 1 Introduction

Over the past 3 years, the benefits of virome analyses from environmental samples to monitor the species (or even strains) present in each geographical region have become clear for the scientific community. Despite the recent advancements in the detection, concentration, and subsequent bioinformatic analyses of these samples (Mastriani *et al.* 2022), several major challenges remain to be addressed. Virome studies from environmental samples pose particular difficulties due to the high presence of contaminants, the low concentration of viral particles, and the substantial proportion of phages and bacteria, making it challenging to obtain sufficient high-quality genomic material to accurately represent the entire eukaryotic virome. Consequently, concentration methods are required, together with amplification or enrichment approaches, to obtain a comprehensive representation of the virome of interest in the environment under study (Rusiñol *et al.* 2020, McClary-Gutierrez *et al.* 2021). These challenges, as well as the impact on health and economy of the recent pandemic outbreak, have led to the launch of several projects aimed at improving our understanding of potential pandemic viruses through the analyses of environmental samples from a One-Health perspective (Sinclair 2019).

Metagenomics approaches based on high-throughput sequencing to assess viral diversity have been shown to provide more comprehensive information than previous analyses using conventional molecular protocols (Donaldson *et al.* 2010, Hjelmsø *et al.* 2017, Martínez-Puchol *et al.* 2020). Several approaches are available for processing the samples depending on the goal of the analysis. The amplicon sequencing approach—such as ARTIC (Quick 2020)—is widely used for describing sequence variability and polymorphisms in a full or partial genome. In contrast, the whole-genome sequencing (WGS) approach enables the characterization of all the genomic material present in a sample (Garner *et al.* 2021). Halfway, when the interest is on a set of species or families that constitute a small fraction of the entire virome, the capture-based approach provides higher sensitivity. This method is based on the design of capture probes selected from a set of genomic sequences of the species of interest (e.g. VirCapSeq-VERT; Briese *et al.* 2015). By using these species-specific probes, the samples are enriched with the targeted sequences by positive selection. In clinical samples, the capture-based metagenomics approach has been proved to achieve a 100- to 1000-fold increase in sequenced reads of interest, reducing the background noise (such as host DNA), and increasing coverage up to 95% (Briese *et al.* 2015).

From a Bioinformatics perspective, there is still room for improvement in the analysis of viruses in environmental samples. Even though viral sequence databases are limited and incomplete, which makes it difficult to assign sequences to viral species (Ghurye *et al.* 2016, Garner *et al.* 2021), bioinformatic tools for virome analysis have been developed with different aims over the past few years. There have been published tools to address, from a viral perspective, certain steps of the workflow. These include tools for quality assessment—CheckV (Nayfach *et al.* 2021)—, for metagenome classification—VirFinder (Ren *et al.* 2017), VirSorter (Roux *et al.* 2015)—, or for functional annotation—VMGAP (Lorenzi *et al.* 2011), vConTACT2 (Zablocki *et al.* 2019)—. Likewise, multiple pipelines to automatically perform full metagenomics data analyses have been developed (see Supplementary Table S1 for further details). Three major groups can be distinguished: (1) First published pipelines were automatized, modular, and oriented to the assessment of the virome in clinical samples, such as the description of gut microbiome. The most relevant tools in this group are VirusSeeker (Zhao *et al.* 2017), ViromeScan (Rampelli *et al.* 2016) and VIP (Virus Identification Pipeline; Li *et al.* 2016). (2) Some web-based user-friendly tools were developed with the aim of facilitating metagenomics data analyses for non-bioinformatician users. These are widely used tools by the virologists, however these tools have some limitations in terms of file size and workflow customization. Most popular tools are CZ ID (former IDSeq; Kalantar *et al.* 2020), and Genome Detective (Vilsker *et al.* 2019). (3) More recently, a group of tools designed for virome analysis from a wider range of matrices that integrate multiple viral-oriented tools has been developed to provide more refined results. These promising pipelines require specialized knowledge for the setup since multiple software environments need to be integrated and can take advantage of containerization for replicability of the analyses. Some relevant pipelines here are ViroProfiler (Ru *et al.* 2023), ViromeFlowX (Wang *et al.* 2024) or ViWrap (Zhou *et al.* 2023).

However, while experimental target enrichment protocols are becoming more popular no standard workflow for the analysis of these datasets is available. The present article aims to improve the computational characterization of viral genomic sequences from environmental samples when the interest is on a defined set of viral species that may appear at low concentrations. The proposed approximation has been developed to complement the corresponding Target Enrichment Sequencing (TES) experimental procedure and to optimize resources consumption. To this end, CAPTVRED (*Capture-based metagenomics Analysis Pipeline for tracking ViRal species from Environmental Datasets*), a specific pipeline for analyzing capture-based metagenomics data, was designed and automated using Nextflow (Di Tommaso *et al.* 2017). The development of the CAPTVRED pipeline is centered on finding an optimal solution at each step and presenting the results in a user-friendly, concise, and comprehensive format. Finally, the efficiency of the PANDEVIR capture panel, a newly developed set of probes for capture-based metagenomics (manuscript in preparation) was evaluated using the CAPTVRED workflow to provide an example of its capabilities.

## 2 Systems and methods

CAPTVRED is implemented in Nextflow, a workflow management tool designed to improve and streamline pipeline automation, reproducibility, and scalability to different computer architectures. This implementation allows automatic control and parallelization of the multiple jobs in the protocol thereby enhancing pipeline robustness and flexibility. Together with the main tool, a configuration module is provided to customize the database based on the provided set of targeted species, from now on referred as *viral candidates*. The modular implementation of the pipeline offers the users higher flexibility, multiple approaches for performing some of the steps, reference database customization based on the dataset characteristics, and easy incorporation of downstream or side complementary modules in the future.

The performance of the CAPTVRED pipeline was tested using a set of 15 samples. Six *simple* simulated samples (“simset”) were generated using as reference the genomes of the 30 *viral candidates* (in this case, species included in the PANDEVIR capture panel), uniformly covered and adding mutation rates of 0%, 1%, 3%, 5%, 10%, and 15%. Six *complex* simulated samples were generated using as reference the same set of genomes together with 120 randomly selected sequences (80 phages and 40 prokaryotic genomes) at the same mutation rates. Finally, three real samples were included in the test set. These samples were collected from sewage, bat guano, and pig lixiviates; to process them, the nucleic acids were extracted, sequencing libraries were prepared followed by the capture protocol, and finally samples were sequenced on an Illumina NextSeq platform (400 M reads). See Supplementary Material for more information on the experimental procedures.

The samples in the test set were processed in parallel using three different automated pipelines: CAPTVRED (with default parameters and databases), Genome Detective, and CZ ID. Both Genome Detective and CZ ID are web-based tools designed for user-friendly viral characterization in high throughput sequencing datasets. Details can be found in the Supplementary Table S2. The total number of species identified from the synthetic dataset was assessed together with their coverage and identity distributions. In both measures threshold was set to 70% to allow fair comparisons across approaches. Precision, recall, and F1-statistic were determined as measures to evaluate and compare CAPTVRED and CZ ID performance.

Finally, the performance of the PANDEVIR capture panel, designed to facilitate the characterization and identification of viral species with pandemic outbreak potential, was evaluated using CAPTVRED. A set of five lixiviate pooled samples from cattle, chicken, pig (two timepoints), and rabbit were sequenced with Illumina NextSeq technology in duplicate, one replicate was processed with standard protocol (WGS) and the other with TES approach. All samples were processed with CAPTVRED pipeline as a single run on a Debian server with 32 threads. Details were described in the Supplementary Material file.

## 3 Implementation

**Figure 1.**
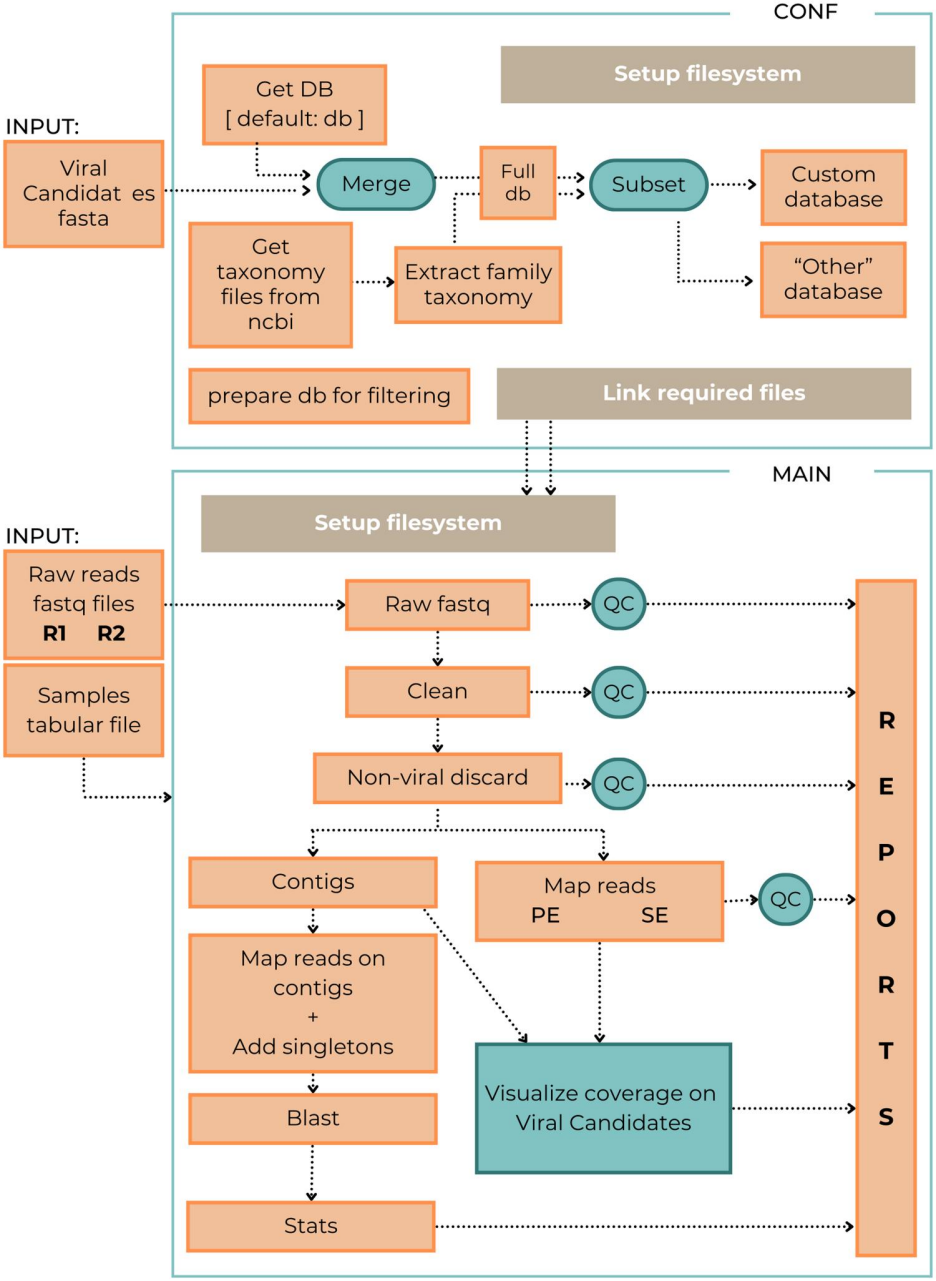
CAPTVRED pipeline workflow overview.

The pipeline is built on four main blocks. (1) Filtering of low-quality and non-viral reads to reduce the computational and time costs. (2) Assembly of reads into contigs, with two available algorithms. (3) Reads mapping and taxonomic assignment, providing three alternative approaches and allowing database customization. (4) Finally, in the results integration and visualization module, the behavior and results of the CAPTVRED analyses are gathered into an HTML report that includes links to the quality and computational reports, summary tables, and description of the assignments found at read, reference sequence, and species levels Supplementary Fig. S1. A workflow overview is shown in Fig. 1, and details of each module are provided in Supplementary Material. In all processes, parameters can be modified by the user, resulting in a customized analysis focused on the requirements of the dataset while still remaining as an automated protocol.

## 4 Results and discussion

CAPTVRED stands out as an adjustable pipeline, allowing modification of many the parameters from the command line, such as *E*-values, coverage and identity thresholds, assembly algorithm, or taxonomy imputation protocol. Through the modular implementation, CAPTVRED offers some extra functionalities not available by any other automatized pipeline: A module specifically designed to address the issue of contamination by undesired known sequences in the sample, a specific focus on the findings related to *viral candidates*, and the option to easily change and optimize the reference database. Altogether, this approach enables customization of the automatized workflow so it can suit specific goals of the analyses or sample characteristics.

As detailed in the implementation section, despite being adequate for the analysis of any Illumina-sequenced metagenomic dataset, CAPTVRED implementation is particularly tailored for capture-based viral metagenomics by using a custom subset of the RVDB database, and by providing a clear report focused on *viral candidates*. This approach ensures that the analyses is limited exclusively on *viral candidates* and other species from the same families, while non-relevant fractions are excluded from the final results. This leads to background noise reduction and facilitates the subsequent results curation. RVDB is a curated non-redundant database of eucaryotic viruses, thus, it is suitable for the study of eucaryotic viruses in complex environments, where an important proportion of the viral fraction corresponds to bacteriophages. However, if the focus is on the phage fraction, the reference database should be changed as described in the tool documentation.

CAPTVRED was benchmarked against two other commonly used metagenomics pipelines: Genome Detective and CZ ID (Supplementary Fig. S2). Both platforms offer a user-friendly web-based interface, allowing easy access for any user. The main characteristics of each protocol are described in Supplementary Table S2. However, the protocols are hermetic and standardized, all parameters are completely fixed and computational cost information is not provided. In the case of CAPTVRED, it was locally run on a Debian server with 32 parallel threads. The analysis of the full set took 547.4 CPU hours.

Further analyses were also conducted on the set of *viral candidates* sequences, ensuring focused results visualization and facilitating their interpretation. The benchmarking results of the fifteen samples in the test set (three capture-based metagenomic samples and 12 synthetic samples) reveal some differences among the presented approaches (Supplementary Fig. S2). To ensure a fair comparison, all the results were filtered at 70% coverage and 70% identity, providing a more stringent evaluation and higher reliability on the biological results.

While CAPTVRED workflow obtained results for all synthetic samples, in the CZ ID approach seven out of 12 samples produced results (four simple and three complex samples), and no results were reported by Genome Detective in any sample due to runtime limit reaching. In all the cases for which results were obtained, the 30 *viral candidates* species included in the panel were properly identified (Supplementary Fig. S2). However, few false positives (FP) were reported either by CAPTVRED and CZ ID in complex samples corresponding to *Coronaviridae* and *Filoviridae* families; CAPTVRED also reported two *Coronaviridae* FP in highly mutated synthetic samples, for which CZ ID did not report any outcome (Supplementary Fig. S3). These results were translated into an F-1 statistic of 0.97 for CAPTVRED vs. 0.94 for CZ ID in the 0% mutation rate sample; 0.98 vs. 0.96 in the 1% mutation rate sample, and 0.94 vs. 0.94 in the 3% mutation rate sample (Supplementary Table S4). Statistics for the simple synthetic samples at 10% and 15% mutation rates and complex simulated samples at 5%, 10%, and 15% mutation rates are not reported by CZ ID, since no contigs were assembled in those cases.

For the real samples (bat guano, sewage, and pig lixiviate) few or no species from *viral candidates* were found across all the approaches. Only two species (SARS-CoV-2 and CoV229E) were detected in the sewage sample by CAPTVRED and Genome Detective after filtering (Supplementary Fig. S3); both species were found by CZ ID too with > 99% of identity and 33% and 46% of coverage, respectively. In addition, bovine coronavirus (BCoV) was reported only by CAPTVRED approach; the species was reported by Genome Detective and CZ ID as well but did not pass the cutoffs (coverage: 97.8% and 18.4%, respectively; identity: 68.47% and 98.8%, respectively). Although the results obtained in these samples are not informative enough for some of the viral candidates, other species from *Coronaviridae* family were detected (Martínez-Puchol *et al.* 2024). These are coherent within the biological context since we do not expect to find most of the potential pandemic viruses of the panel in the analyzed environmental samples.

These results indicate that informative and accurate outcomes are obtained with all approaches. Nonetheless, these can be improved by appropriately adjusting the parameters, which stresses the importance of understanding the workflow and the parameters proposed by each platform, not only to enhance performance but also for a proper results interpretation. While, after filtering, CZ ID reported hits with slightly higher coverage and identity with less dispersion, CAPTVRED reports outcomes for degraded or low-quality sequences. This represents an advantage for the analysis of environmental samples, where nucleic acid molecules are usually more fragmented or have lower quality. According to these results, the CAPTVRED tool can be suitable for viral discovery too, since the use of RVDB as a reference database allows to assign sequences with lower similarity to a family or genus when the species is not identified, providing further valuable information for the user. In addition, if required, the parameters can be adjusted depending on the objectives of the experiments, offering a flexible and customized automated analysis.

In summary, and according to the presented results, CAPTVRED represents a suitable pipeline protocol for the analyses of complex environmental samples and for the identification of viral species present at low concentrations, particularly when combined with a specific capture panel. The scalability of this pipeline to a cloud-based server is feasible since it is implemented in Nextflow.

Finally, the CAPTVRED pipeline was used to assess the PANDEVIR capture panel, targeted to 30 potentially zoonotic viral species Supplementary Table S5. The 10 samples (five WGS and five TES) were processed as a unique run with CAPTVRED. Despite time and resource consumption cannot be discriminated by sample, samples processed with TES produced raw outputs of notably smaller file sizes. Pipeline set up and sequenced data analyses resulted in a straightforward and user-oriented processes. Regarding the performance of the panel, the results show up to 1000-fold change in the number of reads corresponding to *viral candidates* or related species (Supplementary Fig. S4) when using the TES approach. In addition, a reduction of fractions of non interest—such as phages or bacteria for this analysis, as shown in Supplementary Fig. S5—is observed when using the capture-based approach. Therefore, the TES approach combined with a capture-oriented Bioinformatics pipeline leads to better results with less time, storage space, and economical resource consumption. Further details can be found in the Supplementary Material.

## 5 Conclusions

CAPTVRED pipeline provides an automated, reliable, and adjustable protocol specially designed for the analysis of viral capture-based metagenomics datasets. The protocol can be tailored at multiple levels of the analyses depending on the user interests and data requirements, parameters can be modified, databases can be customized for specific purposes, and datasets enriched with any capture panel can be analyzed. While basic skills on the bash terminal would be required to run CAPTVRED pipeline, no programming knowledge is necessary, and many possibilities are available to perform a customized and automated analysis. The final report integrates quality reports, taxonomic assignments, and coverage plots for *viral candidates*, it is presented in a user-friendly format and provides compact, encapsulated results easier to share in a single zipped folder.

In the future, CAPTVRED could be extended to other sequencing technologies, like nanopore sequencing, and further HTML extensions could be developed to provide a more responsive results page. Integrating CAPTVRED into a web server would be desirable. Although computational requirements (CPU and memory) are hard to supply for standard Illumina sequencing runs, the Nextflow implementation should ensure a smooth transition to a cloud-based service.

Further advances in viral characterization and discovery (especially in complex samples) will require deeper knowledge of the proper computational parameters to adapt the current protocols to the changing viral diversity and to facilitate the integration of refined capture-panel designs. The flexibility offered by the Nextflow implementation and the modular construction of CAPTVRED provide a flexible design and confer the potential to incorporate those tools and features that may be of interest in the future.

## Supplementary Material

vbae150_Supplementary_Data
