# Supplementary material for: CAPTVRED: an automated pipeline for viral tracking and discovery from capture-based metagenomics samples
Source: Bioinform Adv. 2024 Oct 8;4(1):vbae150. doi: 10.1093/bioadv/vbae150 (PMC11495672; doi:10.1093/bioadv/vbae150)
Supplement: vbae150_Supplementary_Data [file vbae150_supplementary_data.pdf]

SUPPLEMENTARY MATERIAL

CAPTVRED:
An automated pipeline for viral tracking and discovery from capture-based metagenomics samples

Tarradas-Alemany, Maria[1,2]; Martínez-Puchol, Sandra[2,3], Mejías-Molina, Cristina[2,3]; Itarte, Marta[2,3]; Rusiñol, Marta[2,3]; Bofill-Mas, Sílvia[2,3]; Abril, Josep F[1,*]

1. Computational Genomics Lab; Department of Genetics, Microbiology and Statistics; Universitat de Barcelona (UB), Institut de Biomedicina UB (IBUB), Barcelona, Catalonia, Spain.
2. Laboratory of Viruses Contaminants of Water and Food; Department of Genetics Microbiology and Statistics; Universitat de Barcelona (UB), Barcelona, Catalonia, Spain.
3. The Water Research Institute (IdRA); Universitat de Barcelona (UB), Barcelona, Catalonia, Spain.

* **Corresponding author**: Josep F Abril (jabril@ub.edu)

Source code is available at `github` repository:

`https://github.com/CompGenLabUB/CAPTVRED.git`

Further details about CAPTVRED

Flow management and control

CAPTVRED is implemented in NextFlow, a workflow management tool designed to improve and facilitate pipeline automation, reproducibility, and scalability to different computer architectures. Tasks are distributed into workflows, sub-workflows, and processes connected by channels. This implementation allows automatic control and parallelization of the multiple jobs in the protocol and contributes to pipeline robustness and flexibility.

Databases

CAPTVRED pipeline requires the use of two databases.

- Previous to assembly, a filtering step with Kaiju (v.1.9.0; [Menzel *et al.* 2016]) is performed to discard those reads assigned to non-viral taxons. From the nine databases available in Kaiju repositories CAPTVRED analysis uses *nr-euk* by default, since it comprises all taxonomic groups we are interested in discarding. However, the pipeline offers the flexibility to change the reference database or to use a customized one.
- In the taxonomy assignment step the default database is RVDB (Goodacre *et al.*, 2018), a curated and non-redundant viral database that includes all viral (and virus-like) entries hosted by eukaryotes. In the set up, this database is merged with genomic sequences of *viral candidates* to ensure that all are fully represented. After that, the full database is subseted to obtain a smaller database including all the species taxonomically classified in the families to which correspond the *viral candidates*. The customizations of the database aims to reduce the total size while maximizing the variability among the annotated viral species, resulting in a smaller and more oriented database, which leads to a reduction of the overall time and computational costs, and obviates all sequences of non interest in the final results. RVDB is the default database in the pipeline, however this can be changed according to the user interests.

Workflow description

The input files required to run the pipeline include: paired-end sequencing reads raw sequences (in FASTQ format), a tabular file with samples description, and genomic sequences (gzipped FASTA) of the *viral candidates*. Alternative databases can be defined on the configuration files. The pipeline is implemented in 5 modules described below:

Set Up: A separate module to prepare databases and filesystem is included to offer a user-friendly tool. This module does not need to be rerun if the same capture panel is used for multiple runs analyses.

Reads Cleaning and Filtering: Adapters trimming and filtering of low-quality sequences is performed with BBDuk (v38.96; [Bushnell 2018]), with the aim to reduce the size of the datasets and optimize the downstream steps in the analysis. A second filtering step at read level is performed using Kaiju, a tool designed for fast and efficient taxonomic classification at protein-sequence level. After that, all reads assigned to the "Eukarya", "Bacteria" and "Archaea" domains are discarded from the dataset. The quality of the sequences is computed and summarized for the raw sequences, clean sequences, and filtered sequences sets using FastQC(Andrews, 2010) and MultiQC (Ewels *et al.*, 2016). Reports obtained by this tool can be accessed from the CAPTVRED final report generated at the end of the pipeline.

De-novo assembly: The reads are assembled into contigs to generate longer and more informative sequences. This way, a more comprehensive picture of the captured virome is created. CAPTVRED uses MEGAHIT as default assembler (Li *et al.*, 2016), which provides an excellent balance between accuracy and resource consumption (Vollmers *et al.*, 2017). For a more accurate assembly, the pipeline offers the option to use metaSpades (Nurk *et al.*, 2017; Vollmers *et al.*, 2017). However, it must be considered that this assembler only processes the paired-end reads for the assembly (while MEGAHIT integrates single-end read libraries together with the paired-end ones), which can lead to loss of information, especially in complex or low-quality samples. Regarding general assembly parameters, the minimum length for a sequence to be considered a contig or singleton is set by default to 100 bp intending to be conservative and preserve as much information as possible for the taxonomic assignation step, however, as in many other parameters, users can adjust the minimum length according to their interests.

Taxonomy: Three approaches were considered and made available to assign each sequence to a taxonomic group. By default, a NCBI BLASTN (Altschul *et al.*, 1990) search is performed. The custom database used as reference includes all entries in the RVDB database that constitute families of the *viral candidates*. The default pipeline parameters for the BLASTN (E-value $< 10^{-3}$, identity $> 70\%$, and dbsize $= 5 \times 10^{9}$) were chosen with the goal of maximizing both the accuracy and efficiency of the analysis. Again, users can adjust them all according to their interests. Alternatively, TBLASTX search or Kaiju algorithm can be chosen to determine the taxonomic assignations. Finally, this results are summarized in three tables which can be found in the final report (See results integration and visualisation). The total coverage of contigs over the given reference genomes is calculated, and a coverage plot is generated for each of the sequences on *viral candidates*, where the annotated genomic sequence of reference, the mapped reads, and the contigs are represented. The figures are also available in the final report.

Reads Map: After cleaning, reads are mapped onto custom database using Bowtie to provide robustness to the contigs taxonomic classification. Results are included in summary report and represented in the coverage figures for *viral candidates*.

Results integration and visualization: The CAPTVRED pipeline produces an HTML report summarizing key findings with the aim of facilitating the visualization and interpretation of the results. From this page, the user can access the quality and the computational performance reports. It also includes summary tables for metadata and sequences recovery, as well as all viral assignations found at read

level, sequence level, and species level. Finally, coverage figures for each sample are also included. This report is written in `HTML` and automatically filled with the pipeline outputs, embedding javascript libraries for user interaction (*Bootsrtap v4* and *jQuery*). An example of the final report is provided in Supplementary Figure S1.

Sequence contamination filtering: This additional module is designed to filter and discard contaminant sequences that may be in the sample. It searches for all the contigs mapped to sequences of interest (previously given by the user) and discards them from the dataset. This option is not enabled by default, it must be activated on the command line and the potential contaminant sequences need to be provided. In addition, some parameters for this module can be adjusted on the configuration file.

Experimental processing protocol for environmental samples

For the three real samples, nucleic acids were extracted from the samples and prepared for sequencing; amplification was performed using the Sequence-independent Single-Primer Amplification (SISPA) method (Fernandez-Cassi *et al.*, 2018). First, RNA was retrotranscribed and the cDNA strand was complemented, then all genetic material was amplified by PCR and purified. Libraries were constructed using KAPA HyperPlus Library Preparation Kit (KAPA Biosystems, Roche). Libraries construction consisted of fragmentation, indexing with KAPA UDI Primer mixes (KAPA Biosystems, Roche), and amplification of the dsDNA obtained from SISPA. For the enrichment, libraries were equimolarly pooled, hybridized with the `PANDEVIR` capture probes for 20h, and recovered with magnetic Capture Beads (HyperCap Bead Kit, Roche). Samples were sequenced on an Illumina NextSeq platform (400 M reads).

Each pair of files included in the test set have together less than 2GB to allow easier and faster execution of the analyses over all the tools considered (details related to file sizes and amount of reads are reported in Supplementary Table S3).

Assesmment of PANDEVIR panel performance with CAPTVRED pipeline

`CAPTVRED` was used to evaluate the performance of the `PANDEVIR` capture panel. This panel was designed to facilitate the characterization and identification of viral species with pandemic outbreak potential. The first version, the one tested in this analysis, includes 89 genomic sequences from 30 viral species that show a risk of leading to zoonotic events by infecting humans or closer vertebrate species. Viral species included in the panel are described in Supplementary Table S5.

To assess the performance of the mentioned panel, a set of five samples was processed with different experimental protocols. Lixiviates were collected from trucks after animals corpses transport of cattle (one pooled sample), chicken (one pooled sample), pig (two timepoint pooled samples: March and August), and rabbit (one pooled sample). The set of five samples was processed in parallel with standard metagenomics protocol and capture-based approach. All the samples were processed in a single Illumina NextSeq run and analyzed with the `CAPTVRED` pipeline; default parameters were used except for the `BLASTN` E-value, which was set to 10^{-10} (default being 10^{-3}).

At read level, for each taxonomic domain and pseudodomain (Bacteria, Archaea, Eukarya, Phages and non-phage viruses), the $log2$ of fold change (FC) between proportion of reads assigned by the capture and WGS approaches was calculated to determine the enrichment of the viral fraction (excluding phages) and the reduction of assignations in the rest of the pseudodomains. At contig level, the number of assignations corresponding to *viral candidates* and their coverage were reported and compared between the two methodologies.

The heatmap in the Supplementary Figure S5 shows the $log2$ of the ratio between the percent of reads assigned to each domain or pseudodomain in the WGS and the capture-based approaches. The profiles across the five given samples are not very consistent, which underlines the impact of the samples biological variability, as well as the effect of the presence of contaminants in any processed metagenomics sample. However, some trends are clear. One of the main goals of the capture panel is to reduce the bacteria and bacteriophage portions found in the sample, since both usually comprise the greatest fraction in the metagenomic environmental samples and represent all the sequencing depth at the expense of viral sequences from vertebrate hosts. In the case of the phages, three out of five samples show a reduction in the phage fraction ($log_2(\frac{\%reads\ using\ \texttt{PANDEVIR}\ panel}{\%reads\ WGS}) < -0.2$), while in the bacteria fraction no relevant differences are shown. The Archaea fraction is reduced in three of the samples and enriched in one of them. The Eukarya fraction is enriched in four out of the five samples analyzed.

The performance of the `PANDEVIR` capture panel should not be measured by enrichment or reduction in viral fraction since ist was designed to hybridate only sequences from a few viral species, which in many cases are not expected to be found in analyzed samples, and this could lead to misleading conclusions. To evaluate the performance of the panel at a species level, the number of hits for each species and its coverage are reported and classified by viral families. From the nine viral families included in the panel, only species from *Coronaviridae* family were found. The results show enrichment in either the *Coronaviridae* species included in the panel and those not included in it (Martínez-Puchol *et al.*, 2023). In addition, there is a reduction in the number of sequences assigned to each species in all the families not included in the panel. Supplementary Table S4 shows *Coronaviridae* and *Caliciviridae* families results as a representative example. These findings highlight the effectiveness of the `PANDEVIR` capture panel in capturing and enriching samples with the target sequences and similar ones (e.g., other species within the same family).

As aforementioned, there are a few considerations to interpret the biological results. Environmental samples are diverse in terms of biological variability and presence of contaminants, which can lead to biases in the outcomes and the efficiency of the capture probes. Due to the unknown composition of the samples, the presence of species of interest, and their unknown concentration levels, the assessment of the panel accuracy is not trivial and requires further studies.

In summary, the capture-based approach achieves an important enrichment on the genomic sequences assigned to the set of *viral candidates* and a reduction of the bacteriophage fraction. However, the reduction is not accomplished as expected in the Eukarya and Archaea fractions, this information should be taken into special consideration for the improvement of future versions of PANDEVIR

panel; for instance, the inclusion of a broader set of hosts genomic sequences on the probes design protocol for that panel, which would reduce inespecific hibridation of the probes and, thus, increase the specificity of the panel.

References

Altschul, S. F. *et al.* (1990). Basic local alignment search tool. *Journal of Molecular Biology*, **215**(3), 403–410.

Andrews, S. (2010). Fastqc: A quality control tool for high throughput sequence data. `http://www.bioinformatics.babraham.ac.uk/projects/fastqc/`. Accessed: 2022-10-07.

Bushnell, B. (2018). Bbtools: a suite of fast, multithreaded bioinformatics tools designed for analysis of dna and rna sequence data. *Joint Genome Institute*.

Ewels, P. *et al.* (2016). `MultiQC`: summarize analysis results for multiple tools and samples in a single report. *Bioinformatics*, **32**(19), 3047–3048.

Fernandez-Cassi, X. *et al.* (2018). Viral Concentration and Amplification from Human Serum Samples Prior to Application of Next-Generation Sequencing Analysis. *The Human Virome: Methods and Protocols*, pages 173–188.

Goodacre, N. *et al.* (2018). A reference viral database (RVDB) to enhance bioinformatics analysis of high-throughput sequencing for novel virus detection. *MSphere*, **3**(2), e00069–18.

Li, D. *et al.* (2016). `MEGAHIT v1.0`: a fast and scalable metagenome assembler driven by advanced methodologies and community practices. *Methods*, **102**, 3–11.

Martínez-Puchol, S. *et al.* (2023). Target enrichment metaviromics for cohomprensive surveillance of coronaviruses in environmental and animal samples. *Heliyon*, ***[submmited]***.

Menzel, P. *et al.* (2016). Fast and sensitive taxonomic classification for metagenomics with `Kaiju`. *Nature Communications*, **7**(1), 11257.

Nurk, S. *et al.* (2017). `metaSPAdes`: a new versatile metagenomic assembler. *Genome Research*, **27**(5), 824–834.

Vollmers, J. *et al.* (2017). Comparing and evaluating metagenome assembly tools from a microbiologist's perspective-not only size matters! *PloS one*, **12**(1), e0169662.

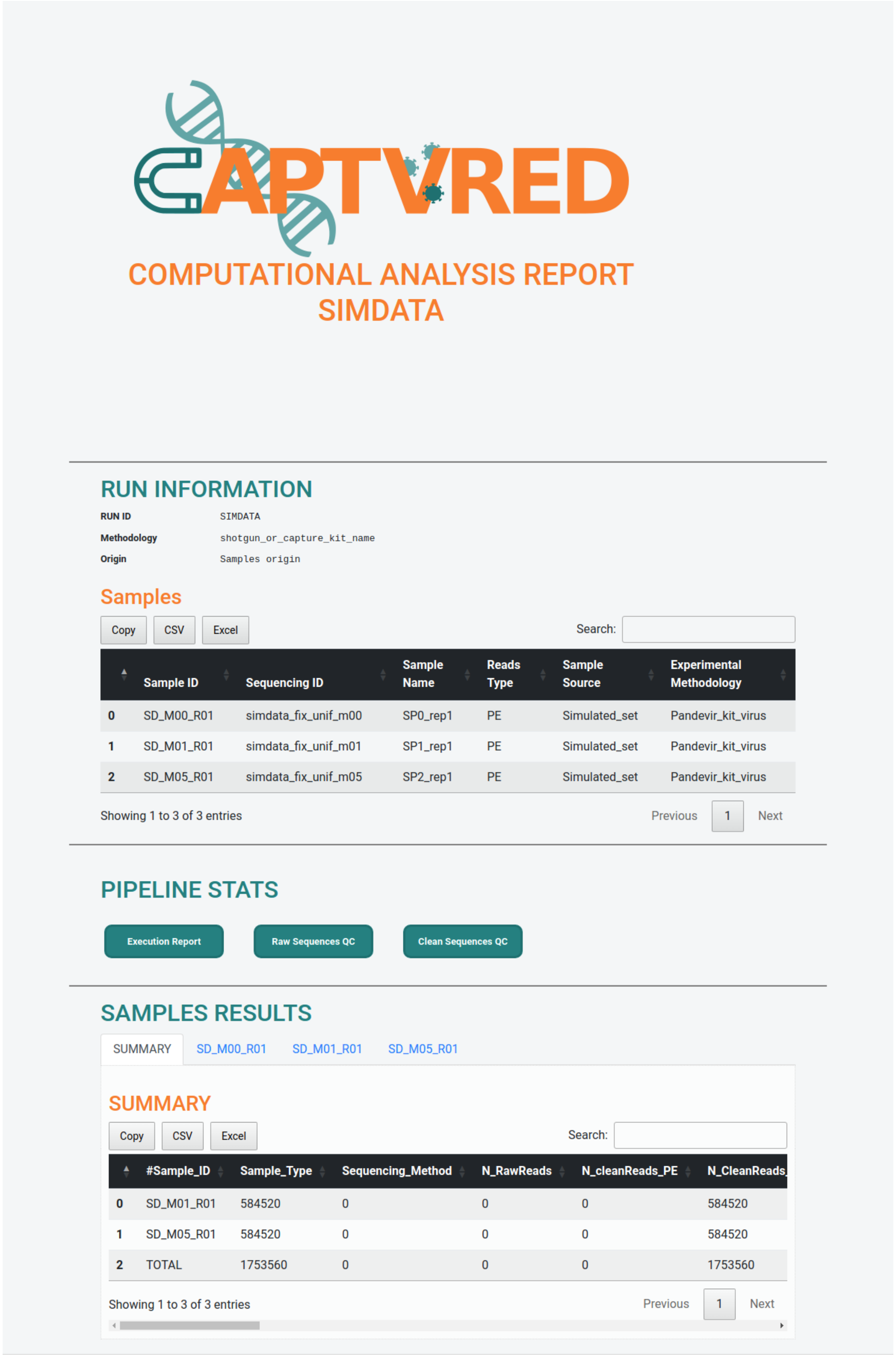

CAPTVRED

COMPUTATIONAL ANALYSIS REPORT
SIMDATA

RUN INFORMATION

RUN ID	SIMDATA
Methodology	shotgun_or_capture_kit_name
Origin	Samples origin

Samples

Copy CSV Excel Search:

	Sample ID	Sequencing ID	Sample Name	Reads Type	Sample Source	Experimental Methodology
0	SD_M00_R01	simdata_fix_unif_m00	SP0_rep1	PE	Simulated_set	Pandevir_kit_virus
1	SD_M01_R01	simdata_fix_unif_m01	SP1_rep1	PE	Simulated_set	Pandevir_kit_virus
2	SD_M05_R01	simdata_fix_unif_m05	SP2_rep1	PE	Simulated_set	Pandevir_kit_virus

Showing 1 to 3 of 3 entries Previous 1 Next

PIPELINE STATS

Execution Report Raw Sequences QC Clean Sequences QC

SAMPLES RESULTS

SUMMARY SD_M00_R01 SD_M01_R01 SD_M05_R01

SUMMARY

Copy CSV Excel Search:

	#Sample_ID	Sample_Type	Sequencing_Method	N_RawReads	N_cleanReads_PE	N_CleanReads_
0	SD_M01_R01	584520	0	0	0	584520
1	SD_M05_R01	584520	0	0	0	584520
2	TOTAL	1753560	0	0	0	1753560

Showing 1 to 3 of 3 entries Previous 1 Next

Fig. S1: **Example of final results report produced by** `CAPTVRED` **pipeline.**

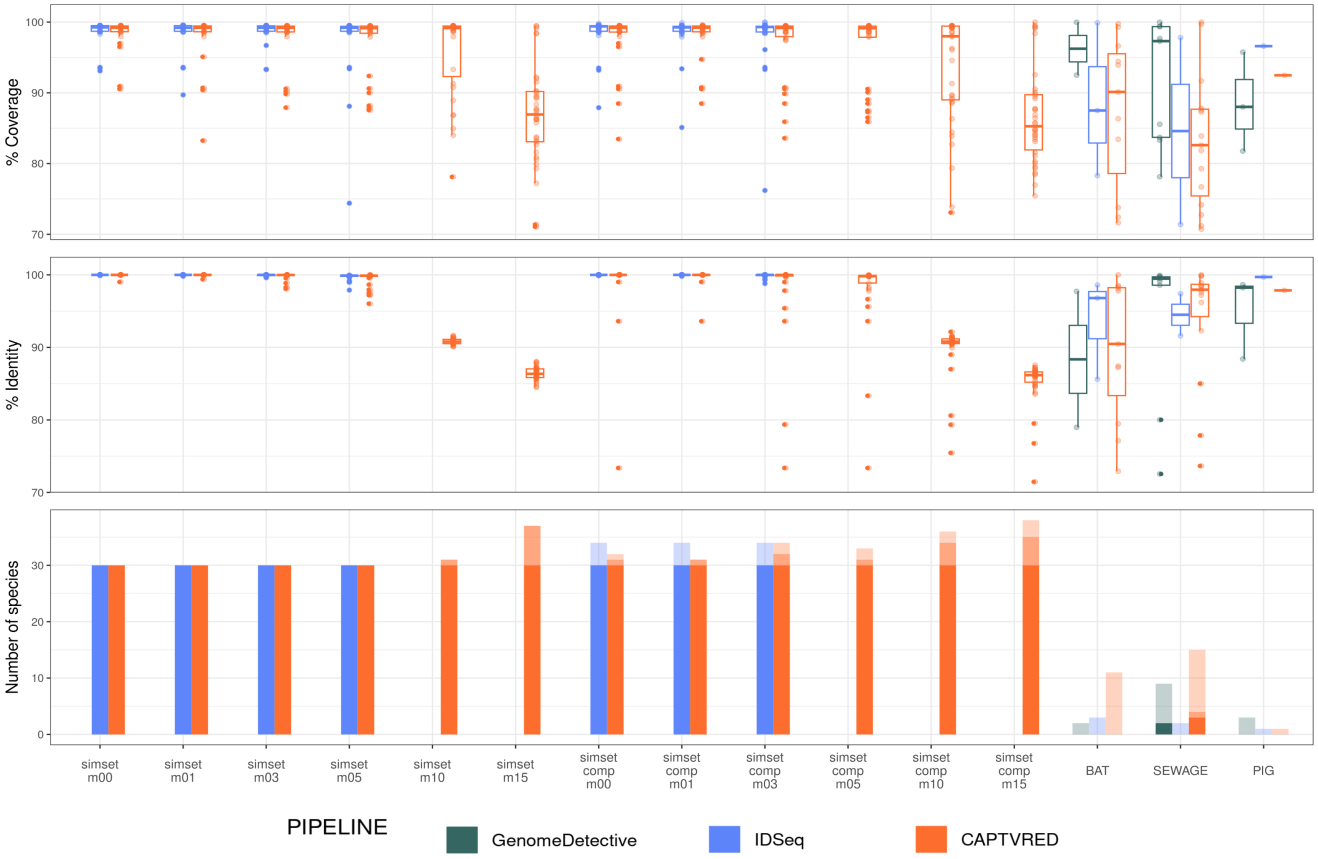

Fig. S2: **Coverage, identity, and total species found benchmarking.** Fifteen samples were analyzed in parallel with three automated pipelines (`CAPTVRED`, `CZ ID` (former `IDSeq`), and `Genome Detective`). Three of them correspond to real samples (bat guano, sewage, and pig lixiviate); six of them to simple simulated datasets ('simset') using as reference the genomes of the *viral candidates* at different mutation rates (0,1,3,5,10, and 15%); and six to complex simulated datasets ('simset comp') using as reference the same genomes together with 120 randomly selected phages and prokaryotic genomes at same mutation rates. Top panel shows the coverage distribution of the species found in each dataset with the three approaches. Mid panel shows the percent of identity distribution of the species found in each dataset with the three approaches. At the bottom panel, the number of species found in each sample is represented, where solid colors show the number of species included in the capture panel, light colors represent the number of related species (species not included in the panel that belongs to one of the nine families present in the capture panel) and pale colors correspond to other viral species. Missing results for some of the tools under comparison are due to runtime limit reaching or results not passing the quality thresholds (explained in detail in the Results and Discussion section).

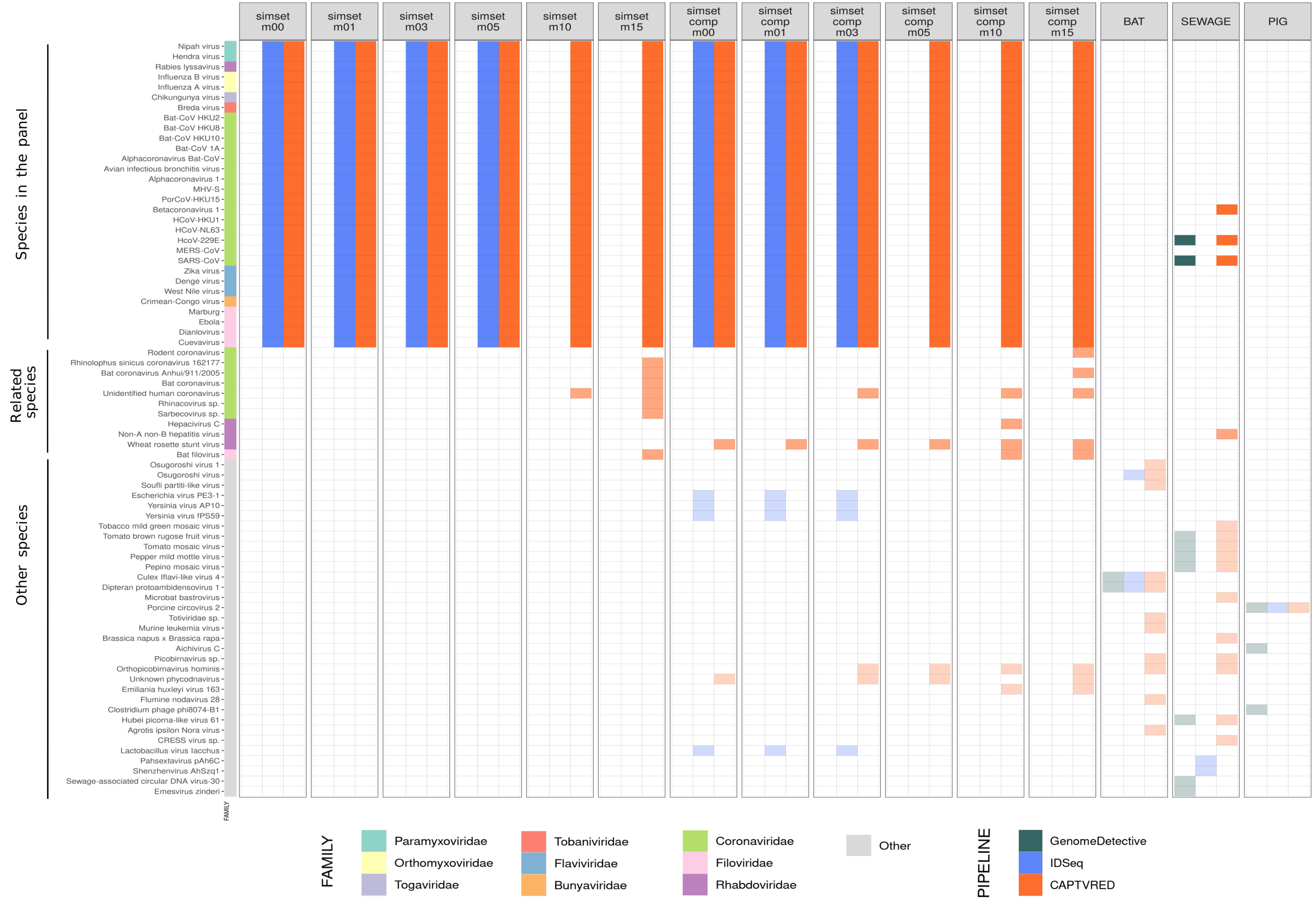

Fig. S3: **Species detected by three automated pipelines.** Fifteen samples were analyzed in parallel with three automated pipelines (`CAPTVRED`, `CZ ID` (former `IDSeq`), and `Genome Detective`). Three of them correspond to real samples (bat guano, sewage, and pig lixiviate); six of them to simple simulated datasets ('simset') using as reference the genomes of the species included in the `PANDEVIR` panel at different mutation rates (0, 1, 3, 5, 10, and 15%); and six to complex simulated datasets ('simset comp') using the same genomes together with 120 randomly selected phages and prokaryotic genomes at same mutation rates. The heatmap shows the species identified in each of the datasets by each of the approaches after filtering by 70% identity, 70% of coverage, and having at least 1 assembled contig. In all cases, when the analysis produces results(see details in Results and Discussion section), all the species included in the panel are found in synthetic datasets. When sequence similarity is decreased related species are more likely to be described and some phages are also reported, yet they are false positive matches.

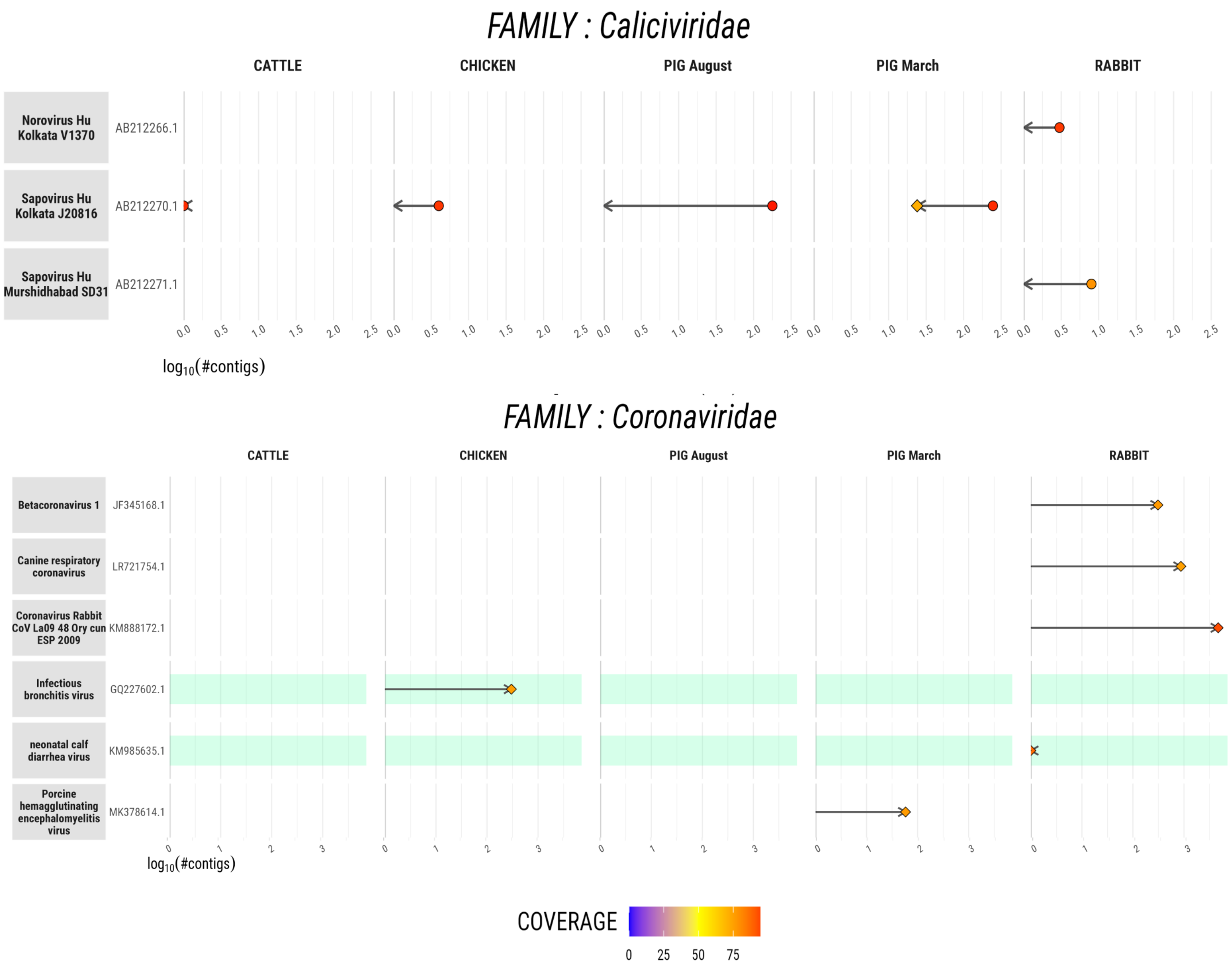

Fig. S4: **WGS vs. capture-based metagenomics approaches**. The number of sequences assigned to each species in WGS (circle) and capture (diamond). *Calciviridae* (top) and *Coronaviridae* (bottom) families are represented in the above plot, each column corresponding to a sample from cattle, chicken, pig, and rabbit lixiviates. Species included in the `PANDEVIR` capture panel are highlighted in green. Shape filling for circles and diamonds is proportional to the query sequence coverage (see coverage color gradient at the bottom legend).

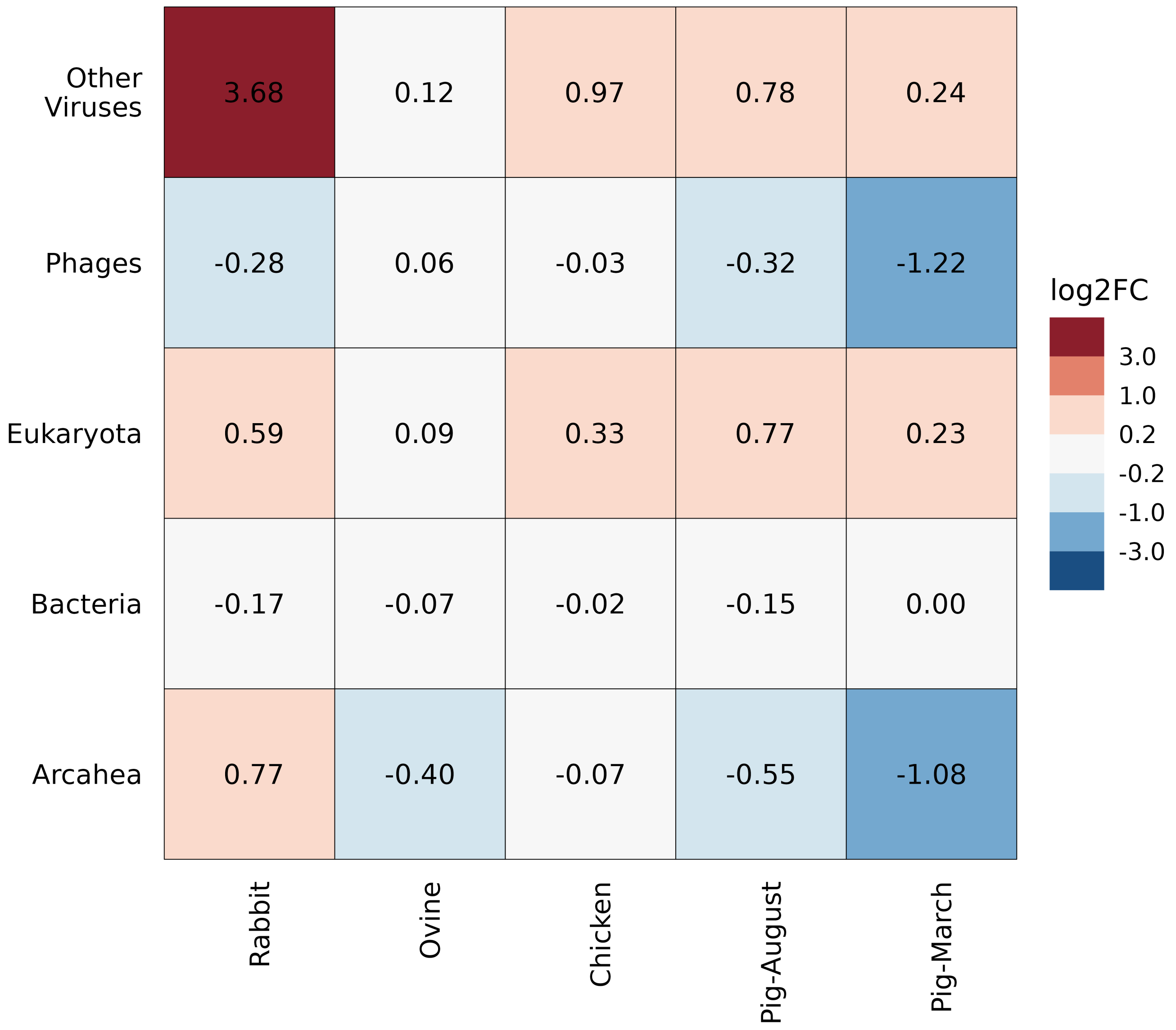

Fig. S5: **Effects of the PANDEVIR capture panel in the proportion of viral and non-viral reads.** The heatmap shows the log_2 of the ratio between percent of reads in PANDEVIR capture approach and the percent of reads in WGS approach for each fraction.

Table S1. **Desctiption of principal charachertistics of pipelines for viral metagenomics data analyses.** Number of citations was obtained from Scopus on july 2024.

NAME	Year	N cit.	Description	Input data required	Sequencing Platform	Implementation	Databases	Target Sample	Repository
ViromeFlowX	2024	0	Viral taxonomic annotation and gene functional analyses. Integrates viral identification tools	Raw fastq reads	Illumina	Docker or Singularity. Nextflow implemented.	UniRef90, eggNOg, NCBIRefseq, crAssphage, pfam, dep. of third-party integrated tools.	Gut microbiota. Tested on faecal metagenomes.	https://github.com/01life/ViromeFlowX/blob/main/docs/usage.md
ViWrap	2023	5	Full stream of virus analyses. Integrates state-of-art tools	Metagenome assembly and raw fastq reads (optional)	-	Conda. Modular implementation.	NCBI Refseq, VOG HMM, custom MAGs db.	Any, including environmental. Not tested in the article.	https://github.com/AnantharamanLab/ViWrap
ViroProfiler	2023	9	Viral metagenomics data analyser. Decontamination option, remove conting redundancy. Integrates viral identification tools.	Raw fastq reads	Illumina	Docker, Singularity or Conda available. Nextflow implemented.	EggNOG, KEGG46, PFAM47, VOGDB, NCBI viral RefSeq, CARD, ResFinder, VFDB5 and dependencies of third-party integrated tools.	Bacteriophage profiling. Tested on 14 simulated samples.	https://github.com/deng-lab/viroprofiler
CZ ID (former IDSeq)	2020	109	Web based tool to characterize microbes present in samples from multiple matrices, including viruses, bacteria, and eukaryotes.	Raw fastq reads and metadata.	Nanopore and Illumina	Web server (Free)	STAR, NCBI-nt and NCBI-nr	Any. Tested on clinical samples for pediatric meningitis pathogen detection and novel coronavirus detection.	https://czid.org/
GenDet	2019	215	Web-based software application. Short read sorting, assembly and annotation.	Raw fastq reads	Nanopore and Illumina	Web server (5 Samples free per new user)	NCBI Refseqs, SWISSPLOT, UniRef90	Any. Tested on synthetic virus dataset.	https://www.genomedetective.com/
VirusSeeker	2017	68	Set of fully automated and modular software package designed for mining sequence data to identify sequences of microbial origin.	Raw fastq reads	Illumina	SLURM Workload Manager	Curated NCBI NT and NR.	Gut Microbiota and clinical samples. Tested on monkey gut samples.	https://github.com/guoyanzhao/VirusSeeker-Virome
ViromeScan	2016	62	Characterize microbiome of complex communities from shotgun metagenomic sequencing datasets.	Raw fastq reads	Illumina	Conda	Eukaryotic Virus Database and Human Virus Database (custom from NCBI).	Intestinal Microbiota. Tested on synthetic samples and Human Microbiome Project data.	https://github.com/simonerampelli/viromescan
VIP (Virus Identification Pipeline)	2016	80	Viral pathogen identification from clinical samples.	Fastq, fasta, SAM or BAM file.	ION Torrent, 454, Illumina	Docker	Custom Human, viral and bacterial databases from NCBI available databases.	Clinical samples. Tested on clinical sample from dengue outbreak	https://github.com/keylabivdc/VIP

Table S2. `CAPTVRED`, Genome Detective **and** `CZ ID` **pipelines comparison.**

FEATURES	CAPTVRED	Genome Detective	CZ ID
**Accesibility**	Open source;<br>commmand line;<br>local	Under suscription,<br>first 10 samples can be freely analyzed;<br>web-based platform;<br>cloud-based	Open source under license agreement;<br>commmand line version<br>and web-based platform;<br>cloud-based
**Input files**	`FASTQ` or `FASTA` format;<br>paired-end reads	`FASTQ` format;<br>Paired-end and single-end reads	`FASTQ` or `FASTA` format;<br>Paired-end and single-end reads
**Maximum input size**	None	2GB	Unknown
**Metadata required**	YES	NO	YES
**Sequencing platforms supported**	Illumina	Illumina, nanopore,<br>Ion Torrent, and Pac Bio	Illumina and Nanopore(*beta*)
**Customizable**	YES (most parameters)	NO	Two alternatively pipelines are available<br>depending o sequencing platform:<br>Nanopore and Illumina are suported.
**Filtering steps**	Non-viral sequences filtering	None	Host-filtering
**Assign thresholds (Default)**	70% Coverage;<br>50% Identity	50% Identity<br>Assuming 45% for uncovered regions	None;<br>User can change thresholds in graphical interface
**Databases used**	NCBInr+euk<br>RVDB<br>*viral candidates* genomic sequences	NCBI Refseqs<br>SWISSPLOT UniRef90	Host specific databases(STAR)<br>NCBI-nt and NCBI-nr
**Simplified workflow description**	1. Clean and filter reads.<br>2. Fast taxonomic classification and discard non-viral sequences(protein level).<br>3. Assembly into contigs.<br>4. Taxonomic assignations against RVDB customized.<br>5. *viral candidates*-specific analyses: map reads and contigs to the genomic sequences of reference.<br>6. Reporting and visualization.	1. Clean and filter reads.<br>2. Taxonomic assignation of the reads and classify in buckets (protein level).<br>3. De novo assembly for each bucket.<br>4. Blast against Refseqs.<br>5. Join contigs for each taxon group.<br>6. Reporting and visualization.	1. Host Filtering and Quality Control.<br>2. Assembly-based alignment.<br>3. Reporting and visualization.
**Source**	`https://github.com/CompGenLabUB/CAPTVRED.git`	`https://www.genomedetective.com/`	`https://czid.org/`

Table S3. **Computational cost on the test sets analyses by each of the approaches.** File sizes, number of sequences in the raw files and running times are reported for each approach. Runtime is reported in CPU hours for the `CAPTVRED` approach and in real time for `CZ ID` and `Genome Detective` since no CPU information is available for these approaches.

SAMPLE	Size(MB)	#Sequences	`CAPTVRED` Runtime (CPU hours)	`CZ ID` Runtime (Total hours)	`Genome Detective` Runtime (Total hours)
SEWAGE	593	7,040,628	204	4h 23'	1h 48'
PIG	1,673	20,055,126		1h 14'	30h 26'
BAT	1,008	13,390,134		1h 41'	1h 32'
Simset m00	150	1,584,350	343.4	0h 58'	-
Simset m01	157	1,584,352		1h 00'	-
Simset m03	169	1,584,354		1h 29'	-
Simset m05	179	1,584,356		1h 44'	-
Simset m10	196	1,584,358		1h 06'	-
Simset m15	210	1,584,360		0h 57'	-
Simset comp m00	1,175	12,392,896		2h 16'	-
Simset comp m01	1,231	12,392,896		2h 19'	-
Simset comp m03	1,328	12,392,896		2h 33'	-
Simset comp m05	1,405	12,392,896		2h 18'	-
Simset comp m10	1,542	12,392,896		2h 16'	-
Simset comp m15	1,647	12,392,896		2h 02'	-

Table S4. **Precision, Recall, and F1-Statistic of synthetic datasets.** True positives (TP, number of hits corresponding to the exact 30 species in the panel), false positives (FP, hits corresponding to any other viral species: related species, other families and phages), and false negatives (FN, species present in the panel but not identified in the analyses) were computed for all the synthetic datasets analyzed with `CAPTVRED` and `CZ ID` (former `IDSeq`). After that, precision ($P = \frac{TP}{TP+FP}$), recall ($R = \frac{TP}{TP+FN}$), and F1-statistic ($F1 = 2 \times \frac{P \times R}{P+R}$) were computed and reported. Both algorithms achieve an F1-statistic over 0.85 which indicates good performance on the test set. No statistics are reported by `CZ ID` for the simple synthetic samples at 10 and 15% mutation rates, nor by the complex simulated samples at 5, 10, and 15% mutation rates, since no contigs were assembled in those cases. `CAPTVRED` has better precision scores since fewer FP are produced.

SAMPLE	PIPELINE	Precision	Recall	F1-statistic
Simset m00	CAPTVRED	1	1	1
	CZ ID	1	1	1
Simset m01	CAPTVRED	1	1	1
	CZ ID	1	1	1
Simset m03	CAPTVRED	1	1	1
	CZ ID	1	1	1
Simset m05	CAPTVRED	1	1	1
	CZ ID	1	1	1
Simset m10	CAPTVRED	0.97	1	0.98
	CZ ID	-	-	-
Simset m15	CAPTVRED	0.81	1	0.90
	CZ ID	-	-	-
Simset comp m00	CAPTVRED	0.94	1	0.97
	CZ ID	0.88	1	0.94
Simset comp m01	CAPTVRED	0.97	1	0.98
	CZ ID	0.88	1	0.94
Simset comp m03	CAPTVRED	0.88	1	0.94
	CZ ID	0.88	1	0.94
Simset comp m05	CAPTVRED	0.91	1	0.95
	CZ ID	-	-	-
Simset comp m10	CAPTVRED	0.83	1	0.91
	CZ ID	-	-	-
Simset comp m15	CAPTVRED	0.79	1	0.88
	CZ ID	-	-	-

Table S5. **Species included in the first version of PANDEVIR capture panel.**

Viral Family	Viral Species	NCBI TaxonId	Sequence description
Filoviridae	Cuevavirus	1513237	Lloviu cuevavirus
	Dianlovirus	2496529	Mengla dianlovirus
	Ebola	186538	Zaire ebolavirus
	Marburg	11269	Marburg marburgvirus
Bunyavididae	Crimean-congo virus	1980519	Crimean-Congo hemorrhagic fever virus
Flaviviridae	West Nile virus	11082	West Nile virus lineage 2
	Dengue virus	12637	Dengue virus 2
	Zika virus	64320	Zika virus
Coronaviridae	SARS-CoV	694009	Severe acute respiratory syndrome coronavirus 2
			SARS coronavirus Tor2
	MERS-CoV	1335626	Middle East respiratory syndrome-related coronavirus
	HCoV-229E	11137	Human coronavirus 229E
	HCoV-NL63	277944	Human Coronavirus NL63
	HCoV-HKU1	290028	Human coronavirus HKU1
	Betacoronavirus 1	694003	Human coronavirus OC43 (HcoV-OC43)
			Bovine coronavirus isolate (BcoV-ENT)
	PorCoV-HKU15	1965089	Porcine coronavirus HKU15
	MHV-S	694005	Murine hepatitis virus
	Alphacoronavirus 1	693997	Transmissible gastroenteritis virus(CcoV)
			Feline infectious peritonitis virus (FcoV)
	Avian coronavirus	694014	Avian infectious bronchitis virus
	Alphacoronavirus Bat-CoV	2492658	Alphacoronavirus Bat-CoV
	Bat-CoV 1A	694000	Bat coronavirus 1A
	Bat-CoV HKU10	1244203	Rousettus bat coronavirus HKU10
	Bat-CoV HKU8	694001	Bat coronavirus HKU8
	Bat-CoV HKU2	693998	Bat coronavirus HKU2
Tobaniviridae	Breda virus	74501	Bovine torovirus
Togaviridae	Chikungunya virus	37124	Chikungunya virus
Orthomyxoviridae	Influenza A virus	11320	Influenza A virus H1N1
			Influenza A virus H2N2
			Influenza A virus H3N2
			Influenza A virus H5N1
			Influenza A virus H7N9
			Influenza A virus H9N2
	Influenza B virus	11520	Influenza B virus
Rhabdoviridae	Rabies lyssavirus	11292	Rabies virus
Paramyxoviridae	Hendra virus	63330	Hendra virus
	Nipah virus	121791	Nipah virus
